# Evaluating AI-based breastfeeding chatbots: quality, readability, and reliability analysis

**DOI:** 10.1371/journal.pone.0319782

**Published:** 2025-03-17

**Authors:** Emine Ozdemir Kacer

**Affiliations:** Department of Pediatrics, Faculty of Medicine, Aksaray University, Aksaray, Turkey; Kalasalingam Academy of Research and Education, INDIA

## Abstract

**Background:**

In recent years, expectant and breastfeeding mothers commonly use various breastfeeding-related social media applications and websites to seek breastfeeding-related information. At the same time, AI-based chatbots-such as ChatGPT, Gemini, and Copilot-have become increasingly prevalent on these platforms (or on dedicated websites), providing automated, user-oriented breastfeeding guidance.

**Aim:**

The goal of our study is to understand the relative performance of three AI-based chatbots: ChatGPT, Gemini, and Copilot, by evaluating the quality, reliability, readability, and similarity of the breastfeeding information they provide.

**Methods:**

Two researchers evaluated the information provided by three different AI-based breastfeeding chatbots: ChatGPT version 3.5, Gemini, and Copilot. A total of 50 frequently asked questions about breastfeeding were identified and used in the study, divided into two categories (Baby-Centered Questions and Mother-Centered Questions), and evaluated using five scoring criteria, including the Quality Information Provision for Patients (EQIP) scale, the Simple Measure of Gobbledygook (SMOG) scale, the Similarity Index (SI), the Modified Dependability Scoring System (mDISCERN), and the Global Quality Scale (GQS).

**Results:**

The evaluation of AI chatbots’ answers showed statistically significant differences across all criteria (p <  0.05). Copilot scored highest on the EQIP, SMOG, and SI scales, while Gemini excelled in mDISCERN and GQS evaluations. No significant difference was found between Copilot and Gemini for mDISCERN and GQS scores. All three chatbots demonstrated high reliability and quality, though their readability required university-level education. Notably, ChatGPT displayed high originality, while Copilot exhibited the greatest similarity in responses.

**Conclusion:**

AI chatbots provide reliable answers to breastfeeding questions, but the information can be hard to understand. While more reliable than other online sources, their accuracy and usability are still in question. Further research is necessary to facilitate the integration of advanced AI in healthcare.

## 1. Introduction

Breastfeeding is the healthiest and most natural food source for newborns and infants and has significant short- and long-term effects on the health of mothers, infants, and young children [1]. Breastfeeding rates vary from society to society. Worldwide, only 44% of newborns are breastfed within the first few hours of life[2]. The reasons for low breastfeeding rates are generally the socio-demographic characteristics of mothers and infants, their social environment, health problems and inadequate breastfeeding education received before and after birth [3].

With the development of technology in recent years, much health-related information has become accessible via the internet. Expectant and breastfeeding mothers commonly use various breastfeeding-related social media applications and websites [4]. Therefore, it should be ensured that expectant and breastfeeding mothers have access to accurate information from digital platforms such as the internet and social media [5,6]. According to a 2019 study, 2.79 billion people worldwide use social media and the internet. However, the usefulness of information is affected by its quality and patients’ ability to understand it [7]. Therefore, it is important to verify the accuracy and usability of internet-based information.

Artificial intelligence (AI) [8], especially its applicability to healthcare, is an exciting area of research today. Humanity aims to create intelligent machines capable of performing tasks that typically require human intelligence, such as problem solving, decision making, and language understanding with AI [9,10]. AI software can serve as a source of information for both healthcare professionals and patients. Many studies have evaluated the effectiveness of AI in areas such as diagnosis, treatment planning, and patient monitoring [11–13].

Despite the growing prevalence of AI chatbots, the potential of these tools in addressing breastfeeding-related health information remains largely untapped. This study addresses this critical gap by focusing on pediatric and maternal health topics within the context of AI applications. Unlike previous studies, which have primarily focused on general medical information or adult health topics, this research offers a novel perspective by examining the unique needs of expectant and breastfeeding mothers.

AI models, such as ChatGPT (OpenAI), Gemini (Google), and Copilot (Microsoft), make information understandable by simplifying complex medical terms. In this way, they facilitate access to information by helping patients and their families understand health issues more easily [14–17]. In addition, the accuracy and reliability of any information from chatbots, not just health-related information, is cause for concern [18,19]. Currently, there are a limited number of studies comparing these chatbots with respect to pediatric topics [20–22]. Therefore, it is important to further investigate and evaluate the role of AI software in healthcare [11].

AI chatbots that can interact with humans using natural language can provide useful information to help with care-related decisions. The goal of our study is to understand the relative performance of such chatbots by evaluating the quality, reliability, readability, and similarity of the information they provide.

## 2. Materials and methods

### 2.1. AI-Based chatbots included in the study

The focus of this study is to evaluate the breastfeeding information provided by three AI chatbots: ChatGPT version 3.5 (OpenAI, 2023), Gemini (Google, 2023), and Copilot (Microsoft Edge, 2023). ChatGPT is a general-purpose chatbot trained on a large database. Gemini is the latest and most powerful AI model introduced with Massive Multitask Language Understanding (MMLU), which can interpret images, text, video and audio, and is multilingual. Copilot uses artificial intelligence and natural language processing technologies to assist users in text-based conversations.

### 2.2. Identifying questions about breastfeeding

**Fig 1:**
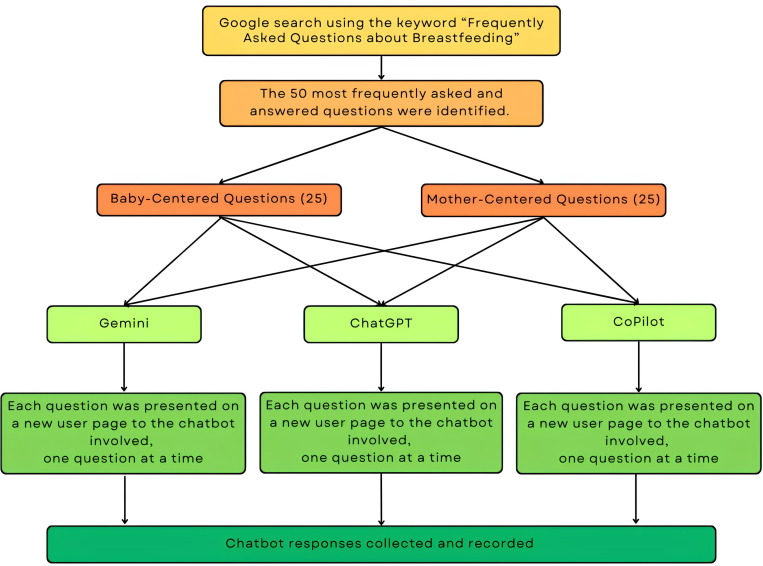
Flowchart of study procedure.

On February 1, 2024, two researchers (E.Ö.K. and İ.K.) conducted a Google search with the keyword “Frequently Asked Questions about Breastfeeding” to identify the most frequently asked questions by non-experts. E.Ö.K. is a pediatrician with 11 years of clinical experience, and İ.K. is a medical doctor with 6 years of clinical experience. Both doctors have at least 4 years of clinical experience in breastfeeding-related areas. The 50 most frequently asked and answered questions were identified. The questions related to breastfeeding are shown in Table 1. The questions were classified into two categories: “Baby-Centered Questions” and “Mother-Centered Questions.” The responses to the 25 questions in each category were evaluated separately. “Baby-Centered Questions” pertain to matters pertaining to breastfeeding and the infant, whereas “Mother-Centered Questions” relate to issues concerning breastfeeding and the mother. Each question was presented on a new user page to the chatbots involved in the study, and they were asked one by one. Table 2 shows some examples of the answers given by the chatbots to the questions asked. All chatbot responses are presented in the Annex, Chatbots’ Responses. The flowchart of study procedure was shown in Fig 1.

**Table 1:** Questions related to breastfeeding.

Baby-Centered Questions	Mother-Centered Questions
1. How often should I breastfeed my baby?	1. Can my nipples crack during breastfeeding?
2. Which positions are best during breastfeeding?	2. What foods should I avoid while breastfeeding my baby?
3. What are the criteria for effective breastfeeding?	3. Can I take medication while breastfeeding?
4. How long should I breastfeed my baby?	4. Can I get pregnant while breastfeeding?
5. How do I know when my baby is full?	5. How often should I change breasts while breastfeeding my baby?
6. Why do babies fight while breastfeeding?	6. What should I do if my baby shows breast rejection during breastfeeding?
7. How often should I wake my baby during the night?	7. What environmental factors should I avoid while breastfeeding my baby?
8. What are the benefits of breastfeeding for the baby?	8. What techniques can I use when breastfeeding my baby?
9. What should I pay attention to when breastfeeding my baby?	9. How often should I change breasts while breastfeeding my baby?
10. How should I support my baby’s head while breastfeeding?	10. Should I get breastfeeding training?
11. What are breastfeeding clothes for my baby?	11. Who gives breastfeeding education?
12. How can I avoid distractions around me while breastfeeding my baby?	12. Can I diet while breastfeeding my baby?
13. What can I do if my baby has gas pains after breastfeeding?	13. How often should I check on my baby while breastfeeding?
14. Will my baby be full if I feed him/her only through breastfeeding?	14. What kind of music can I listen to while breastfeeding my baby?
15. Is it normal if my baby vomits during breastfeeding?	15. Can I lie down while breastfeeding my baby?
16. What should I do if my baby does not want to suck?	16. When should I stop breastfeeding?
17. Which breast should I start from when breastfeeding?	17. How can I relax while breastfeeding my baby?
18. How much weight should my baby gain during breastfeeding?	18. How can I encourage my baby to suck during breastfeeding?
19. What should I do if my baby falls asleep while breastfeeding?	19. How often should I clean my baby’s nostrils while breastfeeding?
20. What should I do if my baby wants to suck more than one breast while breastfeeding?	20. How can I stop my baby from sucking during breastfeeding?
21. What should I do if my baby refuses crying while breastfeeding?	21. How to use plastic nipples for breastfeeding?
22. Why do babies make a squeaking sound when sucking?	22. What should I do if I don’t have a nipple for breastfeeding?
23. Should I worry if my baby’s stool changes color after breastfeeding?	23. How should breast care be after each breastfeeding?
24. What should I do if my baby bites me while breastfeeding?	24. Can the baby be breastfed when the mother is sick?
25. How can I stop my baby from sucking during breastfeeding?	25. What are the benefits of breastfeeding for the mother?

**Table 2:** Some examples of the chatbots’ responses to the questions posed.

	How often should I breastfeed my baby?	Can my nipples crack during breastfeeding?
**ChatGPT**	Newborns: 8–12 times/day; 1–3 months: 7–9 times/day; 4–6 months: 6–8 times/day. Feed on demand and follow hunger cues.	Yes, cracked nipples can occur due to poor latch or dry skin. Use lanolin cream and ensure proper latch to prevent this
**Gemini**	As often as your baby wants, ideally every 2-3 hours during the day and every 3-4 hours at night.	Yes, cracked nipples are a common problem, especially in the early weeks of breastfeeding.
**Copilot**	Typically every 2 to 3 hours, or 8-12 times a day.	Yes, they can crack due to improper latch or dry skin.

### 2.3. Evaluation criteria

Five different scoring criteria were used to evaluate responses to the 25 questions in each category: Quality Information Provision for Patients (EQIP) tool, Modified Dependability Scoring System (mDISCERN), Simple Measure of Gobbledygook (SMOG), Global Quality Scale (GQS), and Similarity Index tool. This scoring criterias were chosen based on their established validity and reliability in assessing written health information [23–27].

The EQIP, developed by Molt et al., is a 20-item scale used to comprehensively assess written medical information [23,28]. The first 14 questions relate to the overall quality of the information in the text, while the remaining questions relate to disease, procedure, or drug information. The EQIP tool includes a rating scale with 4 options: “yes,” “somewhat,” “no,” and “not applicable.” EQIP scores range from 0% to 100%, with higher scores indicating better quality [23].


$$EQIPScore=\left(\left("yes"\times 1+"partially"\times 0.5\right)/\left(20-"notapplicable"\times 1\right)\right)\times 100$$

SMOG is a scale used to predict the level of education required for the average person to understand any text. This scale aims for 100% comprehension [29].


$$\text{Smog Index}=1.0430\times \sqrt{\text{Polysyllable count}\times \frac{30}{\text{Number of sentences}}+3.1291}$$

The reliability and quality of the responses were assessed using the mDISCERN and GQS scales. DISCERN is a brief questionnaire which provides users with a valid and reliable way of assessing the quality of written information on treatment choices for a health problem. mDISCERN is a practical, easy-to-use tool adapted from the original DISCERN five-question reliability tool used in similar scientific studies. It consists of a total of five questions that can be answered “yes” or “no” and scored between 0 and 5 [30]. The GQS scale is a Likert scale developed by Bernard and colleagues to assess the overall quality of the data. It is scored on a scale from 1 to 5 [31]. In our study, these scales were used to evaluate the written texts provided by chatbots.

The similarity of the responses was calculated as a percentage using the Similarity Index scale through the iTenticate program (http://www.ithenticate.com). According to the calculated scores of the responses, 0%–10% indicated high originality, 10%–20% acceptable similarity, 20%–40% high similarity, and over 40% very high similarity.

### 2.4. Statistical analysis

Descriptive statistics, such as maximum, minimum, mean, median, standard deviation, and quartiles between 25% and 75%, were calculated for the collected data. Data that were not normally distributed were evaluated using the Shapiro–Wilk test. The Kruskal–Wallis test was used to determine the mean statistical differences between different chatbots and question categories. Intra- and inter-observer agreement was assessed by rescoring all questions after two weeks and calculating the intraclass correlation coefficient (ICC) with a 95% confidence interval. P values less than 0.05 were considered statistically significant. The Bonferroni multiple comparison test was used and therefore adjusted p-values are given. All statistical analyses were performed using Jamovi software (The Jamovi Project, 2022, version 2.3; Sydney, Australia, available at https://www.jamovi.org).

### 2.5. Ethical approval

This study did not require ethical approval as it did not involve any material obtained from humans or animals.

## 3. Results

The answers given by different AI chatbots were evaluated and categorized according to the given criteria. Statistically significant differences between the chatbots were observed in all evaluation criteria (p <  0.05). Copilot stood out with the highest scores in EQIP, SMOG, and SI scale evaluations (48.9 ±  14.2, 18.5 ±  2.03, and 28.0 ±  20.8, respectively), while Gemini achieved the highest scores in mDISCERN and GQS evaluations (4.15 ±  0.936, 4.12 ±  0.940, respectively). No statistically significant difference was found between Copilot and Gemini for the mDISCERN and GQS scores. The reliability and quality scores showed that all three AI-based chatbots had high reliability and quality. For readability, based on the SMOG index, it was found that they required at least a university education. According to the similarity evaluation results, ChatGPT showed a high level of authenticity (8.56 ±  17.6), while Copilot showed a high level of similarity (28.0 ±  20.8). Descriptive statistics and post hoc comparisons of all categories are shown in Table 3 and Table 4.

**Table 3:** Descriptive statistics and post hoc comparisons of all categories.

General Information and Procedure	ChatGPT3.5 (n = 25)				Gemini (n = 25)				Copilot (n = 25)				*p value*
	Mean ± SD	Median	Min-Max	25%–75%	Mean ± SD	Median	Min-Max	25%–75%	Mean ± SD	Median	Min-Max	25%–75%	
EQIP	42.5 ± 7.60^a^	42.3	22.0 − 55.5	39.1 − 47.9	47.0 ± 9.15^b^	46.5	0.00 − 57.5	42.6 − 52.5	48.9 ± 14.2^c^	52.9	0.00 − 62.6	47.5 − 55.0	<0.001 *
mDISCERN	3.81 ± 0.780^d^	4.08	2.00 − 4.00	3.00 − 4.01	4.15 ± 0.936^e^	4.00	0.00 − 5.00	4.00 − 5.00	4.10 ± 1.23^e^	4.00	0.00 − 5.00	4.00 − 5.00	0.003 *
SMOG	13.3 ± 2.48^f^	14.3	9.30 − 21.2	12.5 − 16.0	16.7 ± 1.93^g^	17.3	11.9 − 20.6	14.9 − 18.0	18.5 ± 2.03^h^	18.8	13.3 − 21.7	17.5 − 19.7	<0.001 *
GQS	3.75 ± 0.714^i^	4.04	3.00 − 5.50	3.00 − 4.00	4.12 ± 0.940^j^	4.00	0.00 − 5.00	4.00 − 5.00	4.10 ± 1.18^j^	4.00	0.00 − 5.00	4.00 − 5.00	<0.001 *
Similarity	8.56 ± 17.6^k^	0.02	0.00 − 10.1	0.00 − 10.0	18.9 ± 17.6^l^	16.6	0.00 − 77.0	0.00 − 30.2	28.0 ± 20.8^m^	27.2	0.00 − 80.0	9.72 − 44.3	<0.001 *

n: Sample Size, SD: Standard Deviation, Min: Minimum, Max: Maximum, 25%: 25 quartile values, 75%: 75 quartile values. EQIP: Ensuring Quality Information for Patients, SMOG: Simple Measure of Gobbledygook, GQS: Global Quality Score. * There is a statistically signiﬁcant difference at p < 0.05.

**Table 4:** Cohen’s d-values of chatbot comparisons of all categories’.

	ChatGPT vs. Gemini	ChatGPT vs. Copilot	Gemini vs. Copilot
EQIP	0,54	0,56	0,16
mDISCERN	0,39	0,28	0,05
SMOG	1,53	2,29	0,91
GQS	0,44	0,36	0,02
Similarity	0,59	1,01	0,47

In the “Mother-Centered Questions” category, only EQIP, SMOG, and Similarity Index showed statistically significant differences between the chatbots (p < 0.001). In terms of reliability and quality, all three chatbots showed high reliability and good quality. The highest mean scores for EQIP, SMOG, GQS, and SI were obtained with Copilot (46.3 ± 13.9, 17.7 ± 2.00, 4.10 ± 1.16, and 26.5 ± 23.0, respectively). The highest mean for mDISCERN was obtained in Gemini (4.47 ± 0.819).  According to the SMOG index, the readability level of all three chatbots was above the university level. According to the similarity score, ChatGPT showed a high level of originality, Gemini showed an acceptable similarity, and Copilot showed a high level of similarity. Descriptive statistics and comparisons of the questions in the “Mother-Centered Questions” category are shown in Table 5 and Table 6.

**Table 5:** Descriptive statistics and post hoc comparisons of the ‘Mother-Centered Questions’ category.

General Information and Procedure	ChatGPT3.5 (n = 25)				Gemini (n = 25)				Copilot (n = 25)				*p value*
	Mean ± SD	Median	Min-Max	25%–75%	Mean ± SD	Median	Min-Max	25%–75%	Mean ± SD	Median	Min-Max	25%–75%	
EQIP	40.5 ± 9.52^a^	41.3	22.0 − 55.0	33.1 − 48.8	45.2 ± 7.13^ab^	46.3	27.5 − 57.5	40.0 − 50.0	46.3 ± 13.9^b^	50.0	0.00 − 62.65	45.0 − 52.5	0.006 *
mDISCERN	4.17 ± 0.791	4.00	2.00 − 5.00	4.00 − 5.00	4.47 ± 0.819	5.00	2.00 − 5.00	4.00 − 5.00	4.13 ± 1.22	4.50	1.00 − 5.00	4.00 − 5.00	0.235
SMOG	13.8 ± 2.58^c^	14.0	9.30 − 2.07	11.9 − 15.5	16.1 ± 1.98^d^	15.9	11.9 − 19.1	15.3 − 17.7	17.7 ± 2.00^e^	17.9	13.6 − 21.9	16.5 − 19.2	<0.001 *
GQS	3.93 ± 0.828	4.00	3.00 − 5.50	3.00 − 5.00	4.27 ± 0.944	4.00	2.00 − 5.00	3.25 − 5.00	4.10 ± 1.16	4.00	1.00 − 5.00	4.00 − 5.00	0.218
Similarity	6.53 ± 9.27^f^	0.00	0.00 − 41.0	0.00 − 9.0	15.2 ± 16.6^fg^	12.0	0.00 − 53.0	0.00 − 27.0	26.5 ± 23.0^g^	20.0	0.00 − 78.0	8.25 − 44.8	<0.001 *

n: Sample Size, SD: Standard Deviation, Min: Minimum, Max: Maximum, 25%: 25 quartile values, 75%: 75 quartile values. EQIP: Ensuring Quality Information for Patients, SMOG: Simple Measure of Gobbledygook, GQS: Global Quality Score. * There is a statistically signiﬁcant difference at p < 0.05.

**Table 6:** Cohen’s d-values of chatbot comparisons of Mother-Centered Questions’.

	ChatGPT vs. Gemini	ChatGPT vs. Copilot	Gemini vs. Copilot
EQIP	0,56	0,49	0,10
mDISCERN	0,37	0,04	0,33
SMOG	1,00	1,69	0,80
GQS	0,38	0,17	0,16
Similarity	0,64	1,14	0,56

Statistically significant differences (p < 0.05) were observed in three of the chatbots’ responses to the 25 questions in the “Baby-Centered Questions” category. All three chatbots showed high reliability and good quality but required university-level training for readability. Copilot had the highest scores for EQIP, mDISCERN, SMOG, GQS, and SI (51.4 ± 14.2, 4.07 ± 1.26, 18.9 ± 1.91, 4.13 ± 1.21, and 29.4 ± 18.6, respectively). According to the similarity index, ChatGPT showed an acceptable level of similarity, while Gemini and Bard chatbots showed a high level of similarity. Descriptive statistics and comparisons of questions in the “Baby-Centered Questions” category are shown in Table 7 and Table 8.

**Table 7:** Descriptive statistics and post hoc comparisons of the ‘Baby-Centered Questions’ category.

General Information and Procedure	ChatGPT3.5 (n = 25)				Gemini (n = 25)				Copilot (n = 25)				*p value*
	Mean ± SD	Median	Min-Max	25%–75%	Mean ± SD	Median	Min-Max	25%–75%	Mean ± SD	Median	Min-Max	25%–75%	
EQIP	44.5 ± 4.40^a^	45.0	35.0 − 52.5	40.0 − 47.5	48.9 ± 0.950^b^	51.3	0.00 − 57.56	47.5 − 55.0	51.4 ± 14.2^c^	55.0	0.00 − 57.5	55.0 − 57.5	<0.001 *
mDISCERN	3.44 ± 0.57^d^	3.55	2.00 − 4.00	3.00 − 4.00	3.83 ± 0.950^e^	4.00	0.00 − 5.00	4.00 − 4.00	4.07 ± 1.26^e^	4.00	0.00 − 5.00	4.00 − 5.00	<0.001 *
SMOG	13.9 ± 2.29^f^	14.5	11.6 − 21.4	13.3 − 16.5	17.22 ± 1.74^g^	17.5	13.5 − 20.4	16.3 − 18.2	18.9 ± 1.91^h^	18.8	13.3 − 21.7	18.1 − 20.0	<0.001 *
GQS	3.57 ± 0.504^i^	4.00	3.00 − 4.00	3.00 − 4.00	3.96 ± 0.928^j^	4.00	0.00 − 5.00	4.00 − 4.00	4.13 ± 1.21^j^	4.00	0.00 − 5.00	4.00 − 5.00	<0.001 *
Similarity	10.5 ± 22.4^k^	0.00	0.00-100	0.00 − 12.0	22.7 ± 18.1^l^	28.0	0.00 − 77.0	8.50 − 30.0	29.4 ± 18.6^l^	29.5	0.00 − 80.0	18.0 − 40.6	<0.001 *

n: Sample Size, SD: Standard Deviation, Min: Minimum, Max: Maximum, 25%: 25 quartile values, 75%: 75 quartile values. EQIP: Ensuring Quality Information for Patients, SMOG: Simple Measure of Gobbledygook, GQS: Global Quality Score. * There is a statistically signiﬁcant difference at p < 0.05.

**Table 8:** Cohen’s d-values of chatbot comparisons of Baby-Centered Questions’.

	ChatGPT vs. Gemini	ChatGPT vs. Copilot	Gemini vs. Copilot
EQIP	1,38	0,66	0,25
mDISCERN	0,50	0,64	0,22
SMOG	1,63	2,37	0,92
GQS	0,52	0,60	0,16
Similarity	0,60	0,92	0,37

According to the averages of the chatbots’ responses to different categories, all three chatbots had higher EQIP scores in the “Baby-Centered Questions” category. When comparing the “Mother-Centered Questions” and “Baby-Centered Questions” categories, statistically significant differences were found for the mDISCERN score in ChatGPT; the EQIP and SMOG scores in Copilot; and the mDISCERN, EQIP, and SMOG scales in Gemini. In terms of reliability, all three chatbots scored higher in the “Mother-Centered Questions” category, but this difference was statistically significant in ChatGPT and Gemini. According to the SMOG scale, the responses of Copilot and Gemini were significantly lower in the “Mother-Centered Questions” category. The data on the responses of the chatbots are shown in Table 9 and Table 10.

**Table 9:** Comparisons of the Baby-Centered Questions and Mother-Centered Questions.

		EQIP	mDISCERN	SMOG	GQS	Similarity
		Mean ± SD	Mean ± SD	Mean ± SD	Mean ± SD	Mean ± SD
ChatGPT3.5	Baby (n = 25)	44.5 ± 4.40	3.44 ± 0.571	13.9 ± 2.29	3.57 ± 0.504	10.5 ± 22.4
	Mother (n = 25)	40.5 ± 9.52	4.17 ± 0.791	13.8 ± 2.58	3.93 ± 0.828	6.53 ± 9.27
	*p value*	0.122	<0.001 *	0.105	0.086	0.781
Gemini	Baby (n = 25)	48.9 ± 0.950	3.83 ± 0.950	17.22 ± 1.74	3.96 ± 0.928	22.7 ± 18.1
	Mother (n = 25)	45.2 ± 7.13	4.47 ± 0.819	16.1 ± 1.98	4.27 ± 0.944	15.2 ± 16.6
	*p value*	0.007 *	0.001 *	0.042 *	0.122	0.109
Copilot	Baby (n = 25)	51.4 ± 14.2	4.07 ± 1.26	18.9 ± 1.91	4.13 ± 1.21	29.4 ± 18.6
	Mother (n = 25)	46.3 ± 13.9	4.13 ± 1.22	17.7 ± 2.00	4.10 ± 1.16	26.5 ± 23.0
	*p value*	<0.001 *	0.607	0.014 *	0.974	0.366

n: Sample Size, SD: Standard Deviation, Min: Minimum, Max: Maximum, 25%: 25 quartile values, 75%: 75 quartile values. EQIP: Ensuring Quality Information for Patients, SMOG: Simple Measure of Gobbledygook, GQS: Global Quality Score. * There is a statistically signiﬁcant difference at p < 0.05.

**Table 10:** Cohen’s d-values of chatbot comparisons of Baby-Centered Questions’ and Baby-Centered Questions’.

	ChatGPT (Baby vs. Mother)	Gemini (Baby vs. Mother)	Copilot (Baby vs. Mother)
EQIP	0,54	0,73	0,36
mDISCERN	1.06	0,72	0,05
SMOG	0,04	0,60	0,61
GQS	0,53	0,33	0,03
Similarity	0,23	0,43	0,14

## 4. Discussion

To our knowledge, this study is the first to evaluate the quality, reliability, and readability of information provided by AI-based chatbots in the breastfeeding domain. In addition, a limited number of studies have evaluated the information differences between AI models and other online information sources [20].

According to our study data, all three chatbots demonstrated high reliability and good quality in their responses. Among the chatbots, ChatGPT provided the most authentic answers, as indicated by its higher originality scores in the Similarity Index evaluation. However, in terms of reliability, Gemini and Copilot outperformed ChatGPT, as they were able to explicitly reference their data sources more consistently. These findings suggest that while all three chatbots are effective in providing high-quality information, their strengths lie in different areas, highlighting the need for further refinement to balance reliability, originality, and transparency across AI platforms.

The Food and Drug Administration (FDA) has approved 521 medical AI models for some medical fields [32]. However, there are currently no regulations governing the use of chatbots for medical purposes. Legal issues that need to be considered when using chatbots in healthcare include source clarity, the impact of misinformation on patient decisions, conflicts of interest, copyrights (e.g., source material(s) chatbots might pull from), and data security.

Fahy et al. [33] discovered notable variations in the reliability of information sources, while Azak et al. [34] reported the low quality and reliability of information in their study. In our study, we evaluated the reliability of three chatbots using mDISCERN, adapted from DISCERN, and found it to be high. Additionally, we observed that Gemini and Copilot were statistically more reliable than ChatGPT. We believe this is because Copilot and Gemini are able to explicitly state the data sources they use.

Many studies based on visual and written data have been conducted in the area of breastfeeding [35]. A literature review evaluating the quality of web-based health information for patients reported significant differences between sources [33]. A study by Azak et al. using DISCERN to evaluate breastfeeding videos found that such videos had high viewership but low quality and reliability [34]. Similarly, Hopkins et al. used the DISCERN tool to evaluate the accuracy and quality of online breastfeeding information and found that 31 websites were included in the study, of which four websites were exemplary [36]. Another study using the GQS scale found that only 18.8% (31 videos out of 165) of breastfeeding education videos were rated as good or excellent. These examples show that videos are a limited resource for patients [37]. Another study emphasized that information provided by physicians or hospitals is of high quality, but educational programs on popular platforms accessible to the public should be developed by experts [38]. In our study, contrary to previous results, we found that the quality of the three different artificial intelligence-based chatbots was good. Thanks to their ability to obtain information directly from the sources in the current literature, we believe that the quality of chatbots’ responses has improved over time and will continue to improve in the future.

A study using SMOG to assess readability in postpartum womefn found that a high level of education was required [39]. Again, a study evaluating the readability of breastfeeding information websites reported that the reading level of these websites was quite difficult and could be understood by university graduates only readily [40]. In our study, the readability of the responses was assessed using SMOG, and it was found that the readability of the responses was difficult, and that they were generally aimed at people with a university education. Based on these data, it is clear that both chatbots and websites target an audience with a certain level of education. Solutions can be developed to improve the readability of the data so that individuals with lower levels of education can easily understand.

Previous research, such as that by Agarwal et al. (2023) and Jedrzejczak and Kochanek (2024), emphasized the potential of AI models in delivering reasoning-based medical knowledge. For instance, Agarwal et al. demonstrated that AI could generate accurate, reasoning-based multiple-choice questions for medical education. Similarly, Jedrzejczak and Kochanek highlighted the variability in chatbot responses in the audiology domain. Our study complements these findings by demonstrating the applicability of AI-based chatbots in breastfeeding, an area previously underexplored.

Nonetheless, there are areas for improvement. As noted by Fahy et al. (2014) and Azak et al. (2023), the reliability and quality of digital health resources are inconsistent. While Gemini and Copilot were statistically more reliable than ChatGPT in our study, concerns about undisclosed sources remain, consistent with Warren et al.‘s (2024) findings on chatbot data transparency.

## 5. Conclusion

Artificial intelligence chatbots demonstrate the potential to provide reliable and high-quality information on breastfeeding. However, the complexity and readability of the information may limit its accessibility to individuals with lower educational levels. While AI-based chatbots outperform many traditional online platforms in terms of reliability and quality, concerns regarding their accuracy, usability, and source transparency remain. Our findings highlight the need for further refinement of AI technologies to ensure that they cater to a broader audience, including those with varying levels of health literacy. Future integration of AI systems into healthcare settings could enhance the accessibility and personalization of medical information. However, continued research is necessary to optimize these platforms and establish guidelines for their clinical and non-clinical use.

*Limitations:* This study has several limitations. First, the evaluations were performed with only three AI platforms, and the response quality of other platforms is unknown. Second, the study may not be fully representative of real clinical scenarios. Third, the chatbots used have different language models, and AI technology is developing rapidly. Therefore, the results may change significantly with updates and new versions. Forth, the same questions weren’t repeated multiple times for each AI chatbot, which may introduce variability in responses. Fifth, at the time of our study, none of the chatbots consistently provided sources for their responses.
